# Familiarity with teammate’s attitudes improves team performance in virtual reality

**DOI:** 10.1371/journal.pone.0241011

**Published:** 2020-10-26

**Authors:** Shannon M. Moore, Michael N. Geuss

**Affiliations:** U. S. Army Combat Capabilities and Development Command, U. S. Army Research Lab, Aberdeen Proving Ground, Maryland, United States of America; University of Milan, ITALY

## Abstract

Virtual reality (VR) is a potentially challenging social environment for effective communication and collaboration. Thus, we conducted a VR study to determine whether increased familiarity with a teammate would improve performance on a joint decision making task. Specifically, because attitude familiarity, or knowledge of another person’s attitudes, has been correlated with better relationship functioning in the past, we anticipated that team performance would improve when teammates were first asked to discuss their task-relevant attitudes with one another. We also hypothesized that increased familiarity would be particularly useful in immersive VR, where typical social and other nonverbal cues were lacking. Twenty pairs recruited from a workplace environment were randomly assigned to either the Familiar or Control condition before completing a joint decision making task both in VR and on desktop monitors. The manipulation of attitude familiarity was successful: pairs in the Familiar condition were significantly more aware of their partners’ unique task-relevant attitudes. Results found that in VR, Familiar pairs were more accurate at determining patterns to events. Additionally, for teams less experienced in VR, Familiar pairs were also more accurate at predicting future events. However, there was no meaningful statistical difference in pairs’ ability to identify information. Familiar teams also took more time to answer questions, and we found no difference in self-reported communication quality. Overall, this was the first successful manipulation of attitude familiarity and results indicate that such an intervention may prove useful in a collaborative work environment, as Familiar teams demonstrated greater accuracy, especially in VR.

## Introduction

As a new social environment increasingly being considered for use in the workplace, it is important to obtain an understanding of the factors that contribute to social success when using virtual reality (VR) to communicate with team members. Previous research suggests that teamwork may suffer in VR [1, 2]. These negative effects may be due to a lack of social norms [e.g., 3] or a lack of feedback, such as traditional communication cues [4, 5]. The absence of social norms and cues could also negatively affect relationship quality, trust, and shared understanding in VR, which work on virtual teams have established as important for team collaboration [6]. In a separate body of work, attitude familiarity, or knowledge of another person’s attitudes, has been linked to smoother interactions with others (e.g., less conflict and more support; [7]) and reports of better relationship quality [8], suggesting that becoming familiar with a partner’s attitudes may facilitate performance in VR where normal social cues can be lacking. The goal of the current research was to investigate whether increasing attitudinal familiarity between team members could improve team performance in VR environments.

### The inherent lack of familiarity in virtual reality

There are several qualities to interactions in VR that suggest a lack of familiarity with one’s teammates could be problematic. Different displays can lead to differences in how people perform [9] or their feelings of presence [10]. The method through which teams interact in VR can vary—ranging from the use of avatars to represent each person to working in the same virtual space with no individual indicators. Traditional communication cues, such as eye contact, smiles, and other nonverbal behavior, which are used to regulate, modify, and control exchanges [4], are not always present during virtual interactions. This is important as performance has been found to suffer in VR when nonverbal feedback is unavailable [5]. Though much of the research on computer mediated communications in the 1990’s refers to text based chatting, we can also apply these lessons to interactions in VR.

We tend to assign others to social categories; but if we do so based on little information, we would likely develop inaccurate expectations about people [11]. This might not only cause interpersonal problems, but these inaccurate expectations could also negatively affect teamwork. The Social Identity Model of Deindividuation Effects (SIDE) stresses the importance of social identity and social context, proposing that they may take on increased impact in computer mediated communications [e.g., 3]. This is due to the fact that such communications are largely more anonymous compared to other forms (e.g., face-to-face), which can lead to a greater emphasis on group identities. Thus, one consequence of the absence of social cues in VR could be the increased tendency to stereotype perceived outgroup members or to wrongly classify others into certain groups. For example, in examining online and offline leisure activities, researchers found that online leisure activities can serve to reinforce gender stereotypes and gendered attitudes, concluding it “perpetuates and even aggravates power relations” [12, p. 262].

Greater feelings of anonymity in VR could also have a negative impact on team interactions. When using computer mediated communications, we may have a decreased awareness of other individuals as well as our own personal identity [13], as feelings of social anonymity can lead people to act more assertively [e.g., 14]. This may be why Kiesler, Siegel, and McGuire [15] found that groups communicating using computers were more likely to make hostile comments or insults. In contrast, when there is greater social transparency, a feeling of accountability exists wherein people become more aware of their self-image and behave more prosocially (e.g., such as in bystander interventions; [16]). Thus, in addition to the difficulty of interacting with others without the social cues and context to which they are accustomed, teammates working together in VR may also have to combat greater feelings of anonymity, which could lead to increased assertiveness or hostility.

### The consequences of situational invisibility

Awareness of situational factors can be difficult as they are often invisible, yet lacking awareness of such circumstances can alter how we perceive others and our relationships. The correspondence bias refers to the tendency to make inferences about a person’s disposition based on behaviors that may also be explained situationally [17]. For example, imagine someone cuts you off on your drive to work. Not knowing anything about them or the situation in which they find themselves, you may assume they are a rude/impatient/unsafe person. In reality, it is possible that you were in their blind spot or that they were unaware their lane was about to end and had to act quickly. Gilbert and Malone [17] described four causes of this bias: lack of awareness, unrealistic expectations, inflated categorizations, and incomplete corrections. In order to recognize the role a situation may play in someone’s behavior, we have to be aware of the situation.

For a team working together, a lack of awareness of one another’s situational or environmental constraints could negatively affect team relations [e.g., 18]. For example, consider a situation where two teammates are working together in VR, unable to physically see one another and viewing information unique from that which their partner sees. If Player 1 is unable to answer a question with his information, he may assume Player 2 is the one with the resources to answer the question. If Player 2 is unable to do so, Player 1 may assume Player 2 is incompetent. It could be the case though that Player 2 is unable to answer the question on his own as well. “It is painfully obvious that observers must be aware of situational constraints if they are to consider the role that such constraints may have played in producing an actor’s behavior” [17, p. 27]. This bias was further illustrated in a study by Cramton et al. [18], who found that when a situational explanation for a partner’s poor performance was not provided, distributed teams were significantly more likely than those in collocated dyads to make dispositional attributions for their partner’s poor performance.

A problem exists in that the types of teams likely to require VR to conduct meetings, such as teammates who work too far away from each other to meet in person or newly assembled distributed teams, are also more likely to be unfamiliar with one another. Thus, the very teams we would expect to truly *need* virtual environments are also the types of teams (e.g., unfamiliar with each other) we might expect to experience difficulty interacting in VR. Given the possible reasons for why team performance could suffer in VR, we suggest a simple intervention below that could be implemented and should help reduce stereotyping, alleviate feelings of anonymity, and may provide more tolerance of different environmental situations.

### Creating familiarity in teams

While the research above indicates that a lack of familiarity with one’s teammate could be detrimental in VR, familiarity itself has been defined in different ways in the literature. Some experimenters have utilized an overall self-report assessment of familiarity, such as when both individuals indicate in self-reports that they know each other “very well” [e.g., 19]. People may be considered “familiar” to the extent that they have known one another for longer periods of time [e.g., 20]. However, perceived familiarity with another individual may not be accurate, and length of relationship is not necessarily indicative of greater knowledge of a person. Below, we will expand on our proposal that a specific type of familiarity, knowledge of others’ attitudes, would be beneficial for team performance—especially for virtual teams. Additionally, we will explore a method to actually create such familiarity, which if successful could be a highly useful intervention.

### Attitude familiarity

While greater knowledge of others in general has been associated with relationship satisfaction [e.g., 21] and more supportive behaviors [e.g., 22], we propose attitude familiarity would be a particularly useful type of partner knowledge. Knowledge of others is beneficial in relationships even when it pertains to their negative qualities [23, 24]. One specific type of partner knowledge is attitude familiarity (AF), or knowledge of another’s attitudes [e.g., 7, 25]. Specifically, attitudes are evaluations of objects, people, situations, events, or behaviors that are stored in memory [e.g., 26]. Attitudes are functional [e.g., 27] guiding behavior [e.g., 28], decision making [e.g., 29], and information processing [e.g., 30]. It has been proposed that AF should enable “individuals to better anticipate, influence, and respond to others’ behavior (i.e., it is functional). The overall effect of attitude familiarity would be to foster relationship processes in daily life” [25, p. 132].

Initial research on attitude familiarity supports the hypothesis that knowledge of others’ attitudes is linked to improved relationship functioning [e.g., 7, 25]. When couples were more familiar with each other’s attitudes, they perceived their partner as more responsive, reported more positive interactions, and had higher state self-esteem compared to less familiar couples [25]. A follow up study found that more familiar couples also reported that they were less likely to fight, less likely to upset one another, more likely to be helpful, and perceived their relationships as more important [7]. Furthermore, attitude familiarity was associated with higher levels of marital satisfaction and satisfaction with life [8]. However, the above work is correlational and conducted exclusively with romantic couples. Thus, in this study, we experimentally manipulated attitude familiarity in an attempt to create better interactions within teams. Just as our own attitudes guide our behavior and decision making, knowing a teammate’s attitudes may allow one to better predict that teammate’s actions and decisions, leading to better team coordination. In a teaming context, greater knowledge of others’ attitudes could translate into improved team performance. In the area of Computer-Supported Cooperative Work (CSCW), there is a relevant focus on familiarity in online collaborative play, as individuals working together can vary from friends to complete strangers, and how the makeup of such teams influences performance or interactions. For example, Nardi and Harris [31] observed that while people may desire alliances where people know each other well in a game, interviews reveal that this can also produce conflict or drama within the teams. Mason and Clauset [32] found that both individual and team performance on a game improved when teams consisted of friends. Teams with more friends (versus strangers) also exhibited more instances of helping behavior and fewer instances of betrayals. This area of work also seems to indicate that greater knowledge of others leads to qualitatively *different* interactions. Our aim, by specifically manipulating attitude familiarity, is to further narrow in on a potential variable that could help teams of strangers work together more smoothly and effectively.

### Why is this work needed?

This work is important because it will help determine if this manipulation—attitude familiarity—can overcome some of the challenges associated with working collaboratively in VR. While past work suggests greater familiarity is useful for new or developing relationships [e.g., 33, 34] there are a few important differences between these works and the current study. Rather than accurate assessments of personality traits between team members, we examined attitude familiarity. Additionally, while these works examined organic first impressions, we manipulated familiarity. There was the real possibility that such an attempt to create knowledge of a teammate’s attitudes could have resulted in a stilted, artificial interaction that does not have the desired effects. However, our goal was to ensure that both teammates were likely to perceive each other accurately. We predicted that those teams assigned to learn each other’s attitudes would perform better than teams in the control condition who did not have a familiarity manipulation.

### The present study

The goal of the current work was to investigate whether familiarity with partners’ attitudes improved team performance in a work setting. To investigate this, we conducted an experiment wherein teams of two were randomly assigned to either learn each other’s task-relevant attitudes or to a control condition where they did not discuss attitudes. For the experimental task, teams worked together both using desktop monitors, a somewhat routine task that allowed face to face interaction, as well as in VR, a more unique experience wherein immediate social feedback regarding one’s partner was more limited. It was predicted that AF would be most useful in work environments that lacked social cues.

We used VR to create a scenario where normative social cues were largely absent. Using each display device, participants performed an information analysis task where they answered questions of varying difficulty as a team. Specifically, they were asked to: 1) identify information on a map, 2) relate multiples pieces of information across players, and 3) predict future events given the information displayed. While partners viewed the same city maps and saw the same focal events, they otherwise had access to unique information about each city. Participants could use a laser pointer to highlight areas of the map for their partner, but they were otherwise unaware of what their partner was viewing and the information he or she had access to. Teammates had a similar operating picture of the environment, but each had access to unique information that required them to fill different roles.

Thus, effective communication was required for partners to work together effectively. It was predicted that because attitudes are functional [e.g., 27], the teams that learned their partners’ task-relevant attitudes would report more correct answers, exhibit faster response times, and report better communication quality compared to those in the control condition. Additionally, it was predicted that attitude familiarity could help mitigate any detriments to team performance that occurred due to the nature of working in VR—a less familiar task wherein normal social cues from one’s partner were minimized.

## Method

### Participants

The CCDC ARL Human Research Protection Program (HRPP) approved the study ARL 18–064. We obtained written informed consent from participants. Participants were civilian government employees or contractors and were required to be 18 years or older, have normal or corrected to normal vision, be able to fuse stereo images, and have normal color vision. No participants were excluded due to prescreening measures. Twenty-four pairs (37 males, 11 females) took part in the study. Participants ranged in age from 18 to 50 (*m* = 27.15, *sd* = 7.49). Twenty-three individuals reported no prior knowledge of their partner, while 25 participants indicated some prior knowledge of their partner. Of those 25, only one individual classified their partner as a “close friend.” One pair was dropped from the analyses, as their response time results were marked as outliers being more than two standard deviations above the mean. We are unable to speculate as to why this pair devoted so much more time to the questions and the overall task compared to others, yet while most pairs completed the study within 2 hours, this pair reached 3 hours and we were forced to end before total completion of all measures due to lab availability.

Additionally, three pairs were dropped from the analyses because their experience in VR was significantly higher than the remaining pairs (on average, roughly 24 times higher than the remaining pairs). Specifically, pair-level VR experience for the 3 excluded pairs was 50, 51, and 100. Including all participants, pair-level VR experience was an average of 12.5 (*sd* = 24). When removing these three pairs, the average pair-level VR Experience was 3.84 (*sd* = 2.89). Due to the wide dispersion (and only a few pairs to represent highly experienced VR users), we purposefully limited our sample to those who had used VR fewer than 20 times.

Once removing these 3 pairs, pairs’ averaged VR experience ranged from 1 to 11.5 instances (*m* = 3.84, *sd* = 2.8). In addition, for each pair, we calculated a difference score for VR Experience to determine whether team members had similar levels of experience. On average, pairs only differed in the number of times they had previously experienced virtual reality by 2.25 instances (*sd* = 1.67) with a range of 0 to 5 suggesting that team-members had roughly equal levels of experience using VR as their partner.

Past research on attitude familiarity found that it predicted relationship outcomes independently of gender (e.g., [7]). Thus, the gender composition of pairs was not the focus of our analyses and we did not assign participants to teams controlling for gender. After 4 pairs were excluded, 12 pairs were male-male, 5 male-female, and 3 female-female. Given the small number of each combination of gender, we did not analyze any effect of gender on results.

A two (Display: Computer Monitor, VR) by two (Familiarity: Familiar, Unfamiliar) design was employed. Display condition was manipulated within subjects, while Familiarity was manipulated between subjects. Participant pairs were randomly assigned to either the Familiar or Unfamiliar condition. The presentation order of the Display condition was counterbalanced across participant pairs (half of the pairs experienced the Computer Monitor condition first while the other half experienced the VR display first). Every pair viewed the same information on all four maps (half viewed in each display condition, counterbalanced across pairs), though Person A always had access to half of the information, while Person B had access to the other half.

### Procedure and measures

Participants who agreed to participate in the study were placed into pairs based on their provided availability. After arriving at the laboratory, all participants completed informed consent and then completed the test of stereo images (Random Dot-E test; [35]), where the letter E is only perceptible in a random-dot stereogram if participants are able to fuse stereo images. Participants were also tested for color blindness (Color Dx test; [36]) and were required to identify shapes drawn in one color but surrounded by other colors, where correctly identifying the shapes indicates participants do not have abnormal color vision. They were then asked if they were hearing impaired. Participants were excluded if they failed any of these three assessments, as doing so would impede their ability to properly view or complete the task. After prescreening, participants were randomly assigned to one of two conditions, where they either learned each other’s task relevant attitudes (“Familiar Condition”) or did not learn each other’s attitudes (“Unfamiliar Condition”).

Participants in the “Familiar” condition completed an icebreaker prior to their shared task, which focused on task-relevant attitudes (see S1 Appendix) as a guide for discussion. They shared with their partners their task-relevant attitudes for a period of 10 minutes. Prior to the discussion, participants were told that the list contained activities or objects relevant to the joint decision making problems they would be doing next. We asked them to discuss their attitudes toward those items with their partner for a period of 10 minutes and specified that they could skip any items they chose, but should limit the discussion to items on the list. They were allowed to be as specific as they wanted, giving background on why they held the attitude or as general as simply indicating whether they liked or disliked an item. They were also told that after the discussion, they would be asked to recall as much as possible about their partner’s attitudes. This was to better ensure that participants focused on the task. After the 10-minute discussion period, participants recorded everything they remembered about the attitudes their partner shared with them for a period of 5-minutes. They were told that their partner would not see their comments. In contrast, participants in the “Unfamiliar” condition began the joint task after the prescreening measures.

For their joint task, pairs answered questions about four different maps which were presented both on computer monitors and in VR. When participants were working in Virtual Reality, they viewed virtual stimuli through an HTC Vive HMD. The resolution within the HTC Vive is 2160 x 1200 with a 90Hz refresh rate and 110° field of view. When participants completed maps in VR, they were seated in desk chairs so that the image portrayed to them (sitting in front of a desk) was aligned with their actual seated position. Participants’ chairs were seated approximately 3 feet away from their partner’s chair. However, with the HMD on, they could not see one another; they could only verbally discuss their answers and thought processes and use the laser pointers to point things out to one another. In contrast, while working on maps on desktop monitors, participants were seated at a table in front of the desktop monitor, while their partner sat directly across from them. Pairs’ desktop monitors were back to back so as not to be able to view one others’ unique variables. Participants were again approximately 3 feet away from one another, but this time facing each other. For these maps, participants could partially view one another over top of the DMs but could also continue to talk and point to items with their partner, similar to in VR.

**Fig 1:**
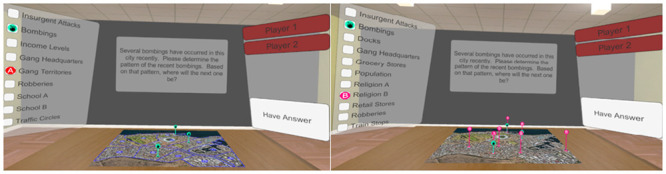
A view of player A’s display in VR compared to player B’s display (Google; imagery: CNES/airbus, Maxar technologies; map data: AfriGIS (Pty) Ltd.).

This map task was intended to serve as a proxy for emergency operations and military mission command. Participant pairs were asked to answer a set of six questions for each of the four maps (See S2 Appendix). Participants had access to the same map and the same data on incidents: recent locations of bombings, attacks, robberies, and assumed gang headquarters. However, additional information differed for each partner (see Fig 1). For example, for one map, Person A also had the option to view economic information, gang territories, locations of school “A”, locations of school “B”, and marked traffic circles. In contrast, Person B had the option of viewing dock locations, grocery and retail stores, population data, the locations of religions “A” and “B”, and train stops (see S3 Appendix—full list of maps and questions). Maps appearing in VR and on the desktop monitors were adapted from Google Maps (see individual images for specific references). All variables added to these maps (e.g., train stops) are fictional content that we created.

Six questions were posed for each map, which consisted of three types of questions.

1. Two questions per map asked the participants to identify information that was presented on one or the other partner’s map.
2. Two questions per map asked the participants to cross reference information presented on each partner’s map.
3. Two questions asked participants to cross reference information presented on each partner’s map and then, as a pair, make a prediction beyond the information presented.

This was then repeated for three different maps, for a total of four maps, with six questions per map. Maps had certain landmarks (rivers, mountain ranges, etc.) in order to specify which part of the map one was focused. Additionally, participants using the computer monitors were able to see the movement of their partner’s mouse, enabling them to point to areas about which they were referring. Participants were always able to view their partner’s mouse throughout working on these maps—both intentional (pointing) and unintentional (selecting items from their menu) actions. Those in VR were also be able to see their partner’s controller, which was held in his/her hand. Participants’ controllers served as a pointer. Similar to the use of the mouse in the DM condition, the controller could be used by the participant to physically draw a circle around an area or point of interest to draw their partner’s attention. Participants’ controllers also served as a laser pointer. When they pressed the button on the controller, a laser appeared that they could use to point to areas on the map more specifically.

A number of factors differ when teams work in VR and on desktop monitors. We chose to utilize each display in the way in which they are typically used. For this early work, we chose to have the actual map and information on it differ only in terms of their ability to utilize the 3rd dimension. Specifically, the only manner in which the display of the maps differed in VR and on the DMs is that maps in VR had 3D components added to them (e.g., the vertical lines reaching upward to mark bombing locations in Fig 1). In this way, the VR displays differed from the DMs in their ability to utilize that 3rd dimension.

**Fig 2:**
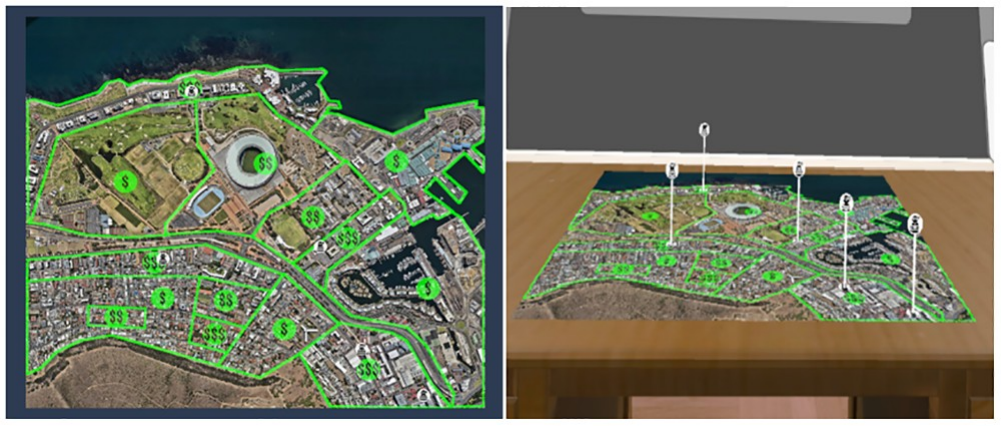
A map as displayed on a desktop monitor, with income level and robberies selected, compared to the same information displayed in virtual reality. (Google; Imagery: CNES/Airbus, Maxar Technologies; Map Data: AfriGIS (Pty) Ltd.).

Participants completed two maps in VR and two maps on the computer monitors while in the same room (see Fig 2). Accuracy was assessed based on whether pairs came to the correct answer (see S3 Appendix for a complete list of questions and answers). We broke down accuracy scores into scores for each of the three types of questions: identifying information, determining patterns to events, and predicting future events based on the information displayed. For response time, we measured the length of time between participants’ indication that they had read each question and the pair’s unanimous decision as to the answer of said question.

Participants were given the following instructions prior to beginning:

“*Now you are going to work with your partner to answer questions about maps that you will view. We are going to show you maps of four different cities. For each map, we will ask you 6 questions. Two of the maps will be examined in VR, while the other 2 will be viewed on computer monitors. In some instances, you and your partner will have the same information (such as where recent events have occurred in the city). But more often, you will have information that is different from one another. You will have to communicate with your partner in order to solve these problems because your partner may have only some or even none of the relevant information. All answers must be unanimous, so you have to clearly convey your relevant information to your partner. You should be as quick as possible in answering the questions while avoiding making mistakes*.”

After each condition, pairs were asked to rate the quality of their communication [37] in VR/computer monitors. Respondents rated the quality of their communication with their partners based on how timely, accurate, adequate, complete, and credible it was. Items were on a 5 point scale where a 1 indicated poor quality and a 5 indicated high quality. A total score was used to determine overall quality of communication. Prior to beginning the task in VR and following each map task in VR, participants were asked to rate their motion sickness (Fast Motion Sickness Scale (FMS); [38]). The FMS requires participants to rate experienced motion sickness on a scale from 0 (no sickness at all) to 20 (frank sickness) verbally. If someone had reported experiencing an increase of more than 10 points, we planned to end their participation in the study, though this did not occur.

Following completion of the map task in both conditions, participants were asked to report their own task-relevant attitudes and their perception of their partners’ task-relevant attitudes. They also filled out relevant measures of video game experience. Specifically, participants were asked to self-report the type of games they normally played and for how long. (See S4 Appendix). Participants were also asked how well they knew their partner from prior interactions (i.e., acquaintances versus close friends) as some pairs may have already known one another. Participants first indicated whether they knew each other prior to the study (yes or no) and then indicated how well they knew each other on a scale of 1 (*Acquaintances*) to 7 (*Close Friends*). Participants were also asked to indicate their sex and age. They were then debriefed. Participants, on average, completed the experiment in about 100 minutes, and the vast majority finished in under 120 minutes.

#### Attitude familiarity

Task-relevant attitudes were assessed by having participants indicate their attitudes toward 27 attitude objects relevant to the task they were asked to complete. These items were selected to represent the behaviors, situations, and objects relevant to the experimental task. Examples of attitude objects in this survey included reading maps, navigating, puzzles, and video games. Participants indicated on a seven-point scale their evaluations of each object, where -3 was *very negative* and +3 was *very positive*. The format of the questionnaire and the scale used were modeled after prior work [e.g., 8]. To assess attitude familiarity, participants also reported what they perceived to be their partners’ attitudes.

Because we are creating attitude familiarity for the purposes of aiding a specific job or task, we chose to have teammates learn each other’s attitudes that were directly relevant to the task that they would complete together. Past work [e.g., 7, 25] examined knowledge of a variety of attitudes toward miscellaneous people, places, and items (e.g., cats, reading, winter, church). While this makes sense, as romantic couples would be in situations where such varied knowledge could be useful, this is likely not the case for teammates in a work setting. Because our goal was to improve performance on a team task, by limiting the attitudes learned to those directly relevant to that task, we not only make the process more efficient, we also increase the chances that teammates will be able to apply such newfound knowledge.

## Results

We found a tendency for familiar pairs to exhibit greater accuracy than unfamiliar pairs. While they spent more time in the decision making phase than unfamiliar pairs, familiar pairs did not self-report better conversation quality.

The goal of the analyses was to determine whether increasing partners’ familiarity with each other’s attitudes improved team performance when conducting an information exchange task in two types of modern information displays (e.g., Desktop Monitor and VR) that varied in terms of the presence of social cues. We quantified team performance using two objective measures: accuracy and response time (RT).

We first report results of a manipulation check that demonstrated participants in the Familiarity condition were indeed more familiar with their partners’ attitudes. Second, we report results that analyze the influence of our familiarity manipulation on multiple measures of team performance. Specifically teams were asked to answer three types of questions that required them to either identify information (type 1), relate information across partners (type 2) or predict future occurrences (type 3). We analyzed how response time (across all types of questions), accuracy for type 1 questions, accuracy for type 2 questions, and accuracy for type 3 questions varied as a function of Display condition, Familiarity condition, and user experience in VR. The same multi-level model was used to evaluate the influence of familiarity in response times (e.g., RTs) and accuracy for type 1, type 2, and type 3 questions. This model included Display condition (Computer Monitor, VR), Familiarity condition (Familiar, Unfamiliar), and Experience in VR included as predictors for each of the team performance measures. Experience in VR was entered in the model to analyze and control for any potential effect of repeated exposure to VR technology that may influence team performance. Display condition, Familiarity condition, and Experience in VR were all entered as fixed effects and Pair Number was entered as a random effect to control for variability associated with Pairs. Display and Familiarity conditions were effect coded and Experience in VR was mean-centered [39]. Experience in VR was averaged across members of each pair.

Multilevel models were conducted using the lme4 package [40] in R version 3.4.3 [41] and restricted maximum likelihood. Multi-level modeling was selected to analyze the data because it allows the inclusion of dichotomous and continuous variables (i.e., VR Experience) in the same model and the ability to assess potential direct and interacting effects of the continuous variable (as opposed to simply “controlling for” VR Experience as would be the case if it were included as a covariate in a traditional repeated-measures analysis of variance). In addition, multilevel modeling controls for dependencies introduced by repeated-measurement (e.g., all participants completed the tasks in both display conditions).

### Attitude familiarity

There are several ways to calculate familiarity. Distinctive indices of familiarity assess whether a person knows how their partner is different from the average person. We calculated distinctive accuracy by subtracting the normative profile (i.e., the average answer per item across all participants) from each partner’s self-reported attitudes as well as each participant’s perceptions of their partner’s attitudes. The resulting correlations between these revised self and informant reports is our measure of distinctive accuracy, indicating the extent that a person is familiar with how their partner’s attitudes are unique from the average person. This is important as it has been suggested that overall indices of familiarity (i.e., correlating raw measures) may be linked to positive outcomes due to the fact that most people report having desirable, normal traits [e.g., 42]. Though we strove to manipulate distinctive familiarity, we also calculated normative familiarity, which is the extent to which a participant perceives their partner as possessing the average person’s attitudes. This was done by correlating participants’ perceptions of their partners’ attitudes with the normative profile. With a lack of specific knowledge of one’s partner (e.g., those in the control condition), we might expect pairs to rely on normative knowledge. We calculated distinctive and normative accuracy to determine if they differed by condition. Past studies of attitude familiarity relied on overall indices, thus it was important to extend the work to distinctive indices as well.

To ensure that our ice-breaker manipulation significantly increased partner’s knowledge of each other’s distinctive attitudes, we conducted an independent samples t-test, where our groups were the Familiar and Unfamiliar conditions, with familiarity calculated as described above and averaged within pairs. We then examined whether distinctive and normative familiarity differed between those conditions. Examining distinctive familiarity, the Familiar condition (*m* = 0.42, *sd* = 0.18) was significantly more accurate in their assessments of their partners’ task-relevant attitudes compared to the Unfamiliar condition (*m* = 0.18, *sd* = 0.21) (*t*(18) = -2.784, *p* = 0.012, Hedges’s *g* = .96 (for a small sample size; [43]). This indicates that we successfully manipulated distinctive knowledge of a partner’s task-relevant attitudes. Findings also suggest that as a result of lacking the specific knowledge of their partners’ task-relevant attitudes, those in the Unfamiliar condition were more likely to rely on normative accuracy when estimating their partners’ attitudes, though this was not significant (*t*(18) = 1.410, *p* > 0.05).

### Accuracy for type 1 questions

Type 1 questions required partners to identify information. For example, “How many low-income neighborhoods are on the map?” These types of questions did not require partners to communicate with each other. In addition, correct answers were of a specific numeric value (e.g., 5 low-income areas). To analyze accuracy on these types of questions, we calculated the absolute difference between the correct answer and the answer provided by participants. Absolute difference was used because there was no meaningful interpretation to over or underestimating. Larger values indicate greater inaccuracy. Results from the multilevel model indicated no significant effects of any factors (see Table 1 for full model results). A non-significant effect of familiarity with a partner’s attitudes on accurately answering these questions is not surprising as only one partner had access to the information to answer this question, and thus collaboration was minimal.

**Table 1:** Full model results for type 1 accuracy.

	Beta	Std. Error	Df	t-value	p
Intercept	1.33	.49	152	2.70	.008**
Display	-.51	.49	152	-1.03	.305
Familiarity	.51	.49	152	1.04	.300
VR Experience	-.23	.26	152	-.88	.383
Display x Familiarity	-.27	.49	152	-.55	.583
Display x VR Experience	.02	.26	152	.09	.931
Familiarity x VR Experience	.07	.26	152	.27	.786
Display x Familiarity x VR Experience	.10	.26	152	.38	.708

Note.

* p < .05,

** p < .01,

*** p < .001. The intercept is representative of the grand mean.

### Accuracy for type 2 questions

Type 2 questions required partners to converse with each other and cross-reference information to determine patterns to events. For example, “Recently, a series of robberies have occurred in the city. Please determine the pattern to these recent robberies.” This type of question required participants to report which variables they believed to be related to a specific event (e.g., robberies were occurring at train stops in high-income areas). Two pieces of information were always 100% spatially correlated with, appearing at and only at the location of, the outcome of interest. A ‘Hit’ was recorded when participants selected one of these spatially correlated variables and a ‘False Alarm’ when they selected a variable that was not spatially correlated with the outcome of interest.

Because pairs could select from several different variables, some of which were related to the outcome and others that were not, we computed a measure of pairs’ sensitivity (d’)—the ability to correctly identify relevant information while correctly ignoring other information. d’ was calculated using the following formula [44], where H indicates number of correct selections or hits and FA indicates the number of false alarms or selections that were not related to the outcome:

d’=ln{[H(1–FA)]/[(1–H)FA]}

Higher d’ values indicate greater sensitivity (e.g., the ability to correctly identify important variables and ignore distractor variables). d’ values were submitted into the multilevel model above to assess the influence of display condition, attitudinal familiarity, and experience in VR.

**Fig 3:**
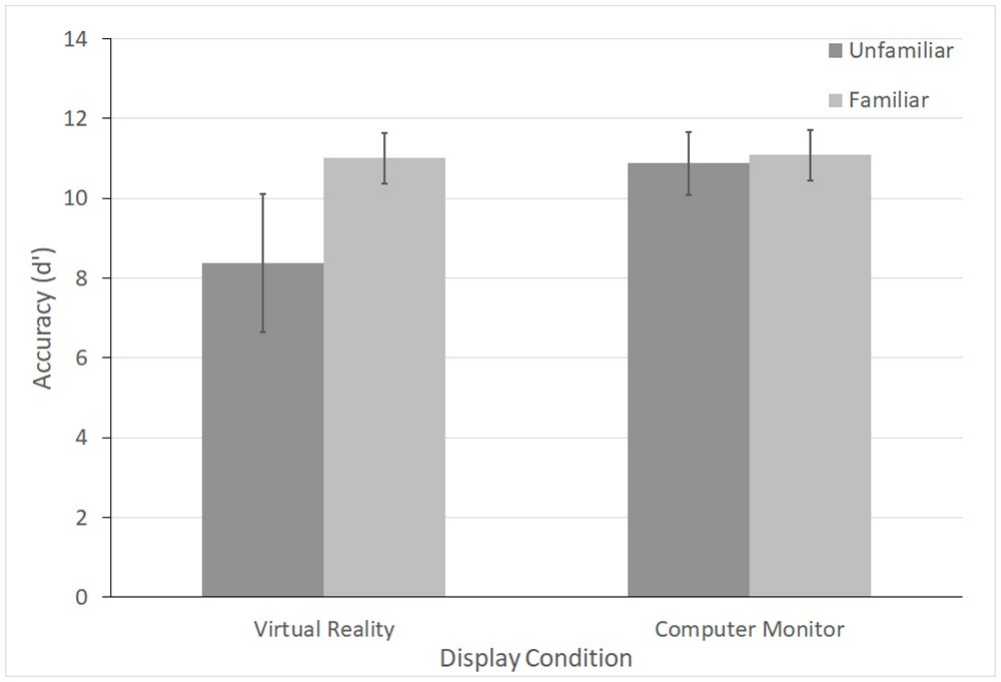
Accuracy for type 2 questions. d’ is plotted for each Familiarity by Display condition. Larger d’ values indicate that pairs were better able to select relevant information while simultaneously ignoring distracting information. Error bars represent standard error of the mean. In VR, familiar pairs performed better than unfamiliar pairs.

There was a significant effect of Display Condition, *β* = -1.096, *S*.*E*. = 0.475, *p* = 0.023, and a significant interaction between Display Condition and Familiarity condition, *β* = 1.328, *S*.*E*. = 0.475, *p* = 0.006. The interaction was largely driven by an effect of Familiarity when participants were in VR. In VR, teams in the Unfamiliar condition (*m* = 8.38, *sd* = 1.73) were less discriminating when selecting relevant variables than those in the Familiarity Condition (*m* = 11.01, *sd* = 0.63). There were no differences between Familiarity and Unfamiliarity conditions (*m* = 11.08, *sd* = 0.64 and *m* = 10.89, *sd* = 0.79, respectively) in the Computer Monitor condition. The results suggest that in VR, an environment with limited social cues, familiarity with partner’s attitudes can improve teams’ ability to correctly identify relevant information while ignoring irrelevant information. See Fig 3. See Table 2 for full model results.

**Table 2:** Full model results for type 2 accuracy.

	Beta	Std. Error	df	t-value	p
Intercept	11.39	.81	19	14.08	1.67e-11***
Display	-1.10	.48	133	-2.30	.023*
Familiarity	.04	.81	19	.06	.957
VR Experience	.45	.43	19	1.05	.305
Display x Familiarity	1.33	.48	133	2.79	.006**
Display x VR Experience	-.45	.25	133	-1.79	.075
Familiarity x VR Experience	-.67	.43	19	-1.58	.131
Display x Familiarity x VR Experience	.32	.25	133	1.26	.211

Note.

* p < .05,

** p < .01,

*** p < .001.

### Accuracy for type 3 questions

Type 3 questions required pairs to work together to determine patterns to events by cross-referencing information, just as in the Type 2 questions. However, to answer the question, they then had to find a location on the map that fit the pattern of those events, but had not yet been targeted. The experiment was designed so that only one location existed that satisfied requirements. Accuracy was calculated by determining first whether participants placed a marker within 12 pixels of the true location, and then averaging across questions to get a percentage of correct trials. Such questions required collaboration as each partner had access to only one of the two variables that constituted the pattern.

**Fig 4:**
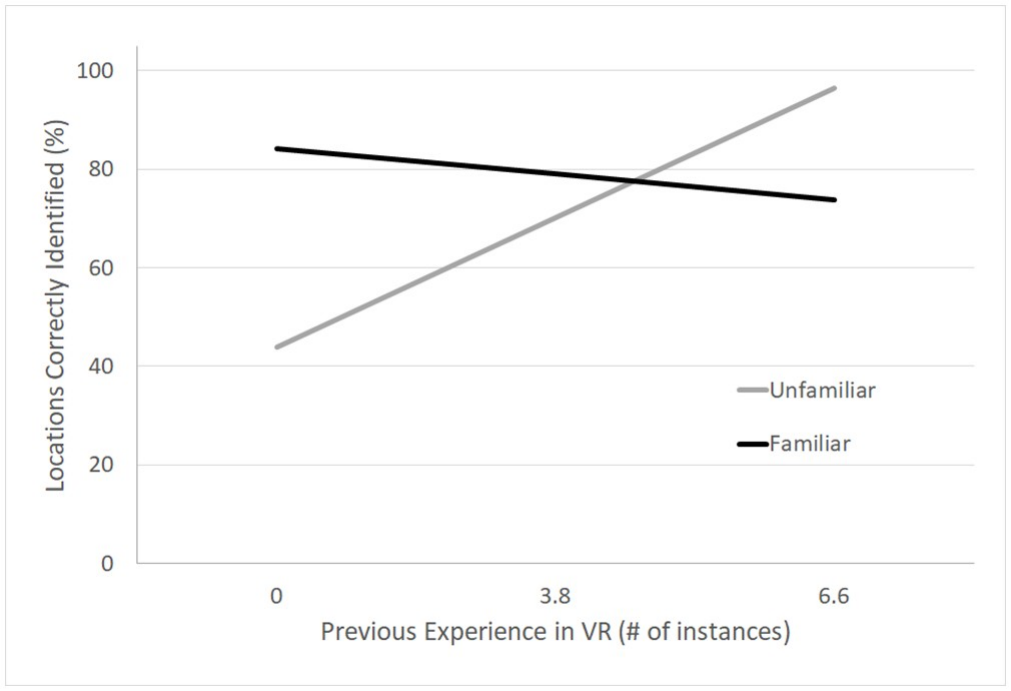
Accuracy for type 3 questions. Predicted values for the percentage of locations correctly identified are plotted against experience in VR for pairs in the Familiar and Unfamiliar conditions. For pairs with little experience in VR, familiar pairs performed better than unfamiliar pairs.

The results of the model revealed a main effect of familiarity, *β* = 0.202, *S*.*E*. = 0.087, *p* = 0.022, that was moderated by a trending interaction between the Familiarity condition and Experience in VR, *β* = -0.056, *S*.*E*. = 0.031, *p* > .05, see Fig 4. For teams with minimal experience in VR (1 SD below the average VR experience, 1), those teams in the Familiar condition (m = 84.25%) identified more locations of future events correctly than teams in the Unfamiliar condition (m = 44.85%). (Expected means are reported given the interaction with VR experience, a continuous variable). This effect of familiarity on performance was not significant for teams with more experience in VR (greater than 3.8 times). These results suggest that there may be a benefit to being familiar with one’s partner when analyzing and predicting future events from shared knowledge. See Table 3 for full model results.

**Table 3:** Full model results for type 3 accuracy (centered at no experience).

	Beta	Std. Error	df	t-value	p
Intercept	.64	.09	152	7.36	1.11e-11***
Display	-.03	.09	152	-.39	.694
Familiarity	.20	.09	152	2.32	.022*
VR Experience	.04	.03	152	1.21	.230
Display x Familiarity	-.02	.09	152	-.23	.820
Display x VR Experience	.01	.03	152	.28	.781
Familiarity x VR Experience	-.06	.03	152	-1.80	.074
Display x Familiarity x VR Experience	.02	.03	152	.51	.611

Note.

* p < .05,

** p < .01,

*** p < .001.

### Response time

**Fig 5:**
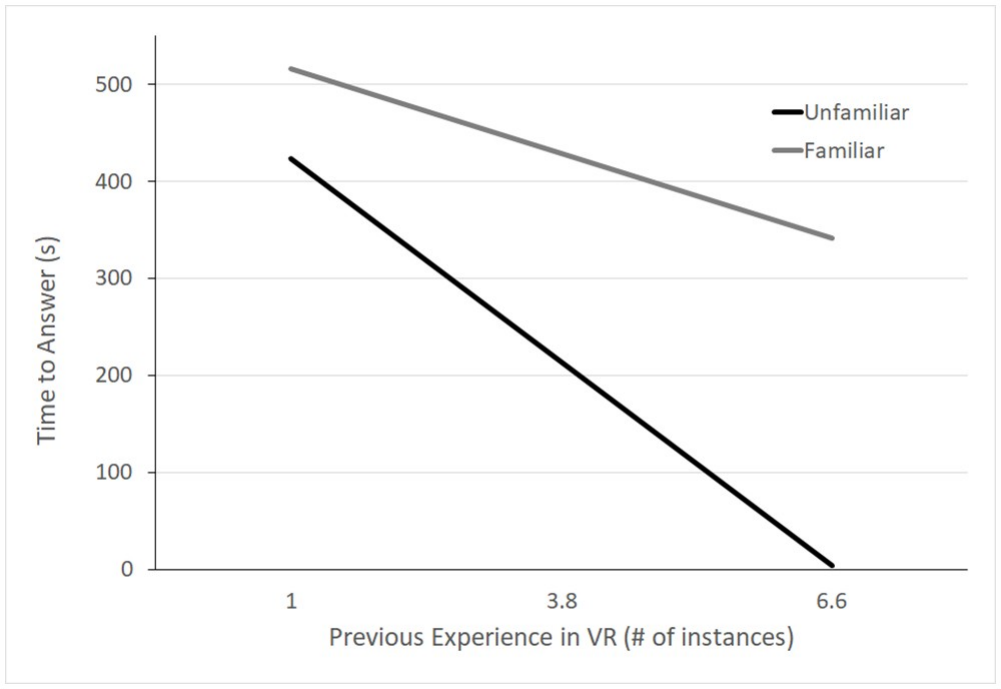
Response time. Predicted response times are plotted for both Familiar conditions with Experience in VR on the x-axis. Familiar pairs took longer to respond than unfamiliar pairs and this difference was exaggerated for pairs with more experience in VR.

Response time was defined as the time between teams indicating they had read the question and indicating they had an answer. Response times for all questions were averaged and entered into the multilevel model described above. There was a main effect of familiarity condition, *β* = 107.58, *S*.*E*. = 32.87, *p* = 0.004, and experience in VR, *β* = -53.06, *S*.*E*. = 19.92, *p* = 0.015. Teams in the familiar condition (*m* = 377.1 sec, *sd* = 158.1) were slower, on average, to answer questions than participants in the unfamiliar condition (*m* = 301.2 sec, *sd* = 87.01). On average, the model predicted that for each additional instance that a team experienced VR, they were about 53 seconds faster to answer questions. Experience in VR, in this sample, ranged from 1 to 12, with an average number of times in VR of 3.84 (*sd* = 2.8). There were no other significant effects or interactions (see Table 4 for full model results). See Fig 5.

**Table 4:** Full model results for response time.

	Beta	Std. Error	Df	t-value	p
Intercept	321.06	32.87	18.78	9.77	8.61e-09***
Display	28.61	27.89	93.31	1.03	.308
Familiarity	107.58	32.87	18.77	3.27	.004**
VR Experience	-53.06	19.92	18.88	-2.66	.015*
Display x Familiarity	-1.98	27.88	93.31	-.07	.944
Display x VR Experience	17.06	16.92	93.53	1.01	.316
Familiarity x VR Experience	21.92	19.92	18.88	1.10	.285
Display x Familiarity x VR Experience	-20.97	16.92	93.53	-1.24	.218

Note.

* p < .05,

** p < .01,

*** p < .001.

### Communication quality

We also examined whether communication quality could be a mechanism through which familiarity influences performance. To test this, we regressed Communication Quality onto the Familiarity condition to see whether familiarity with one’s partner altered reported quality of communication with that partner. Communication quality was calculated using the total score of participants’ self-reported communication quality [37]. There was no significant influence of Familiarity on reported Communication quality, *β* = -0.32, *S*.*E*. = 0.32, *p* > 0.05, suggesting that the mechanism through which Familiarity alters performance is unlikely due to an effect of the perceived quality of communication among team members.

## Discussion

As virtual reality increases in popularity as a way for distributed teams to meet and work together, it is important to acknowledge potential barriers to its successful use, especially because there is evidence that interactions in VR function differently [e.g., 2]. Unfortunately, the very teams that would most need VR are also likely less familiar with one another—increasing the likelihood that they would experience difficulty working together effectively using VR. It has been proposed that just as one’s own attitudes are functional, knowledge of others’ attitudes would be similarly functional—allowing one to better predict and anticipate others’ behavior [e.g., 25]. We predicted that teams that were randomly assigned to learn their teammate’s task-relevant attitudes would exhibit better performance on the task, faster response times, and report better communication quality compared to teams in the control condition. We examined performance in both VR and using desktop monitors, predicting that due to the more invisible nature of teammates’ situation, attitude familiarity might be particularly useful for teams when in the virtual environment, mitigating some negative effects of VR. The results of our study found that even though Familiar teams were slower to respond, they were also more accurate, particularly in VR.

### Familiar teams were more accurate

The utility of task-relevant attitude familiarity to improve team behavior was evident in team performance measures. Overall, the results of this experiment indicate that teams were more accurate when they learned their partners’ task-relevant attitudes. Familiar pairs showed greater accuracy for two of the three types of questions: determining patterns to events when in VR and, for pairs less experienced with VR, predicting future events. Thus, it may be that Familiar pairs spent more time answering questions because they were collaborating more, leading to greater accuracy. This fits with the abundance of literature referencing the speed-accuracy tradeoff (e.g., [45–48]). Specifically, this is the idea that when decision-accuracy is emphasized, people take longer to make the decision and, when decision-speed is emphasized, people are more error prone. As we explicitly instructed participants to “be as quick as possible in answering questions while avoiding making mistakes,” it stands that they perceived the goal as striking a balance between the accuracy and speed. It is possible that being familiar with one’s partner shifted those pairs’ decision-making criteria to prioritize accuracy at the cost of speed or created an atmosphere where familiar pairs felt more comfortable taking longer to achieve better performance. Just as our own attitudes are functional [e.g., 27], it appears that knowledge of others’ attitudes is similarly functional. Past work showed that attitude familiarity was correlated with more positive interactions and impressions of the relationship [e.g., 7, 8, 25]. This is the first experimental work that supports Sanbonmatsu et al.’s [25] supposition that greater knowledge of others’ attitudes may enable us to better anticipate, influence, and respond to their behavior. In hindsight, it also makes sense that improved performance was not evident for questions that involved identifying information. For those items, only one person in the pair had the necessary information. Thus, other than agreeing to let one’s partner answer the question, no collaboration was truly necessary; meaning any benefits of attitude familiarity would not have come into play. Prior literature and our findings suggest that while attitude familiarity in teams would benefit the overall interactions and performance of the team—it likely would not affect one person’s solitary work.

While both display conditions involved aspects of situational invisibility (i.e., a lack of awareness of the information partners’ had access to), this was heightened in VR. As we described earlier, there can be an inherent lack of familiarity in VR. In this instance, pairs could not see each other and therefore were not able to use social cues. Research suggests such a situation could contribute to decreased awareness of self and others [13]. With little information to go on, we may rely on stereotyping [13] or inaccurate expectations [11]. The increased lack of awareness of one’s partner in VR could also be why we witnessed familiarity improving the ability to determine patterns to events in VR, but not on desktop monitors—*because there is a greater need for familiarity in VR*. Why did only those pairs less experienced in VR see an improvement in their ability to predict future events? It may be that due to some participants’ frequent use of VR, that they would be able to more easily adjust to the uncertainty and lack of information about one’s partner (e.g., their expressions, their actions, their access to information) compared to those for whom VR was a more novel experience. This could be why their performance did not improve when they were more familiar with one another. For times when pairs are at a disadvantage (e.g., less familiar with one another), interacting in an environment where there are fewer social cues, or working together at a task they have little experience with, familiarity appears to be one way to overcome these barriers.

### Communication quality in VR

It is surprising that even though Familiar pairs showed evidence of more accurate performance, this was not reflected in participants’ perceptions of communication quality. Familiarity had no influence on reported communication quality. It would be reasonable to expect that teams that performed better were also communicating with one another more effectively. After all, the task required collaboration. Additionally, teams in the Familiar condition were typically slower to answer questions than the Unfamiliar condition, implying longer conversations took place prior to making decisions.

It may be that the utility of attitude familiarity is reflected in behavior, but not in measures of relationship or interaction quality, such as communication. That is, if increased knowledge of others’ attitudes is beneficial by enabling us to better anticipate and respond to their behavior [25] perhaps this advantage is only reflected in participants’ actions (and thus reflected in performance). Past correlational work linked attitude familiarity to reports that couples were less likely to fight or upset one another, and more likely to be helpful [7]. Fights and helpful actions are behaviors.

Yet, other links to attitude familiarity have included perceiving partners as more responsive, reporting more positive interactions [25], and perceiving the relationship as more important [7]. These are fairly global assessments of long-term relationships. It may reason that the relationship benefits of attitude familiarity do not manifest so quickly as to positively influence ratings an hour after attitudes are learned. Another possibility is that the benefits of attitude familiarity are reflected only in overall, global assessments of relationship or interaction quality—not any one rating of a single interaction. Regardless, while self-reported communication quality did not benefit from the familiarity manipulation, pairs did spend more time in the discussion period of the task prior to answering questions, showing increased response times.

### Limitations and future directions

While this study was an important first step toward establishing experimental evidence that a brief manipulation to *increase* attitude familiarity can lead to better teamwork and interactions, we do need to note several limitations that should be addressed by future work. We were only able to recruit a small sample size in this office environment. Additionally, while we used a within design for the display questions, familiarity was manipulated between subjects. Future studies should be conducted using larger sample sizes to further establish whether attitude familiarity can lead to better team performance. Steps should also be taken to recruit participants with a wider breadth of VR experience, such that those with routine VR exposure do not constitute a few outliers as they did here. Another limitation of this work was the lack of an activity for participants in the Unfamiliar group. In future works, pairs could be asked to discuss a topic unrelated to task-relevant attitudes to help rule out the possibility that any type of 10-minute interaction prior to the joint task would be beneficial. However, a topic must be carefully chosen. It would have to be certain that pairs would not stray into discussing any of their attitudes relevant to the main task. This is an important next step though, as we cannot rule out that other types of interactions would also benefit team performance in the ways witnessed here. We proposed that the discussion of attitudes would help better anticipate, respond to and predict teammates’ behavior. Yet, this work is not conclusive.

Other questions arise from this work as well. The role of experience in VR is unclear. While Familiar pairs were more accurate at predicting future events, this only was true for those with less VR experience. VR experience clearly plays a role, yet what that role is remains unclear. It could be that a lack of familiarity and a lack of experience in VR were two barriers that existed for teams in this study. Thus, when both barriers were present (e.g., inexperienced and unfamiliar teams), teams are least effective.

Another question is why attitude familiarity is only reflective in team behaviors, such as performance, and not in interpersonal outcomes, such as communication quality. We must note that our assessment of communication quality in this work was a self-report measure. Future work could utilize audio recordings of teams’ interactions in order to have an objective record of the discussion that took place. This could then be coded and analyzed to determine if Familiar and Unfamiliar teams have qualitatively different discussions. Indeed, future studies overall would benefit from the addition of nonself-report measure. For example, collecting data on heart-rate or other measures that would help draw more objective conclusions regarding relationship quality. Though we found no connection between attitude familiarity and interpersonal outcomes in this work, this could be further investigated by measuring other types of interaction quality. Relatedly, it may be that the benefits of attitude familiarity manifest in terms of relationship quality over time. If an intervention could be applied and studied in long-term teams, researchers could study how attitude familiarity affects team performance and relationship quality over time. It would also be interesting to assess whether attitude familiarity is beneficial for teams only during early teamwork and whether Unfamiliar teams are able to “catch up” in terms of performance as they naturally become more familiar with their teammates over time. Conversely, it could also be that Familiar teams always continue to have an advantage over Unfamiliar teams. As the first experimental manipulation of attitude familiarity and the first application of attitude familiarity to the workplace, many questions remain that must be further explored.

## Conclusions

This is the first successful experimental manipulation of attitude familiarity in the laboratory, following several correlational studies [e.g., 7, 25]. It is also the first work to extend the study of attitude familiarity to nonromantic pairs. Specifically, we attempted to determine whether attitude familiarity would be useful for an entirely different group of people—teams in the workplace. The majority of people have jobs where they must collaborate or work effectively with others. Thus, if this was found to be a successful manipulation for teams, task-relevant attitude familiarity would be useful to the majority of businesses and organizations. For example, in the Army, it is imperative that in human autonomy teams, teammates be able to understand one another’s reasoning and predict their behavior so that the team functions effectively. We foresee attitude familiarity as a way to facilitate smooth interactions within human autonomy teams. Measures of attitude familiarity could be applied as assessments of team potential, as information that enables autonomy to better predict the needs and capabilities of the team, or familiarity could be acquired as a way to quickly create rapport and understanding among new teammates.

Our manipulation check found that we did successfully create distinctive knowledge of partners’ attitudes. Thus, the manipulation we used could be suitable for future studies. Furthermore, this initial test of attitude familiarity found that teams that learned each other’s task-relevant attitudes performed more accurately on questions that required team collaboration. Specifically, they were better at determining patterns to events when working in VR, and for teams less experienced with VR, they were better at predicting future events across both displays. Attitude familiarity is an intriguing concept—by becoming familiar with others’ attitudes, can we better predict and respond to their behavior [e.g., 25], just as our own attitudes predict our behavior [e.g., 28]? While further work is needed, our initial results indicate the answer is yes.

## Supporting information

S1 Appendix (DOCX)

S2 Appendix (DOCX)

S3 Appendix (DOCX)

S4 Appendix (DOCX)
