# Supplementary material for: Familiarity with teammate’s attitudes improves team performance in virtual reality
Source: PLoS One. 2020 Oct 26;15(10):e0241011. doi: 10.1371/journal.pone.0241011 (PMC7588115; doi:10.1371/journal.pone.0241011)
Supplement: S1 Appendix — (DOCX) [file pone.0241011.s001.docx]

S1 Appendix

**Task-Relevant Attitudes**

memory games

icebreakers

participating

in studies

word problems

puzzles

video games

history

working with

a partner

working with a

team

politics

computers

virtual reality

geography

reading maps

independent work

collaborative work

meetings over the

phone

in-person meetings

working early in

the day

working in the

afternoon

working in the

evening

navigating

deadlines

current events

offensive

language

*(i.e., swearing)*

visual displays

of information

information provided

in written form
