# Supplementary material for: Familiarity with teammate’s attitudes improves team performance in virtual reality
Source: PLoS One. 2020 Oct 26;15(10):e0241011. doi: 10.1371/journal.pone.0241011 (PMC7588115; doi:10.1371/journal.pone.0241011)
Supplement: S2 Appendix — (DOCX) [file pone.0241011.s002.docx]

S2 Appendix

Cape Town Map


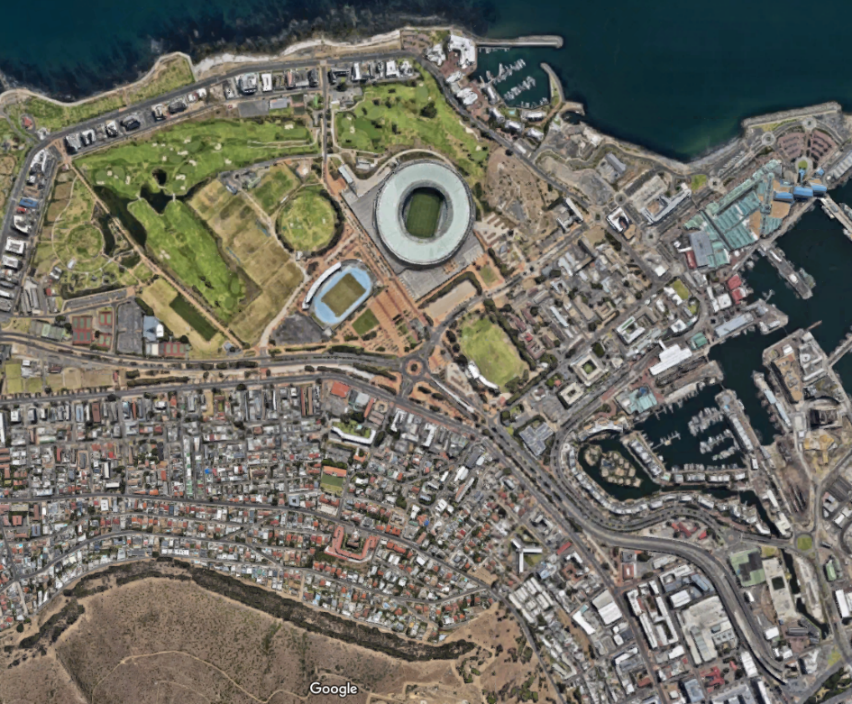


(Google; Imagery CNES/Airbus, Maxar Technologies; Map Data: AfriGIS (Pty) Ltd.).

Tokyo Map


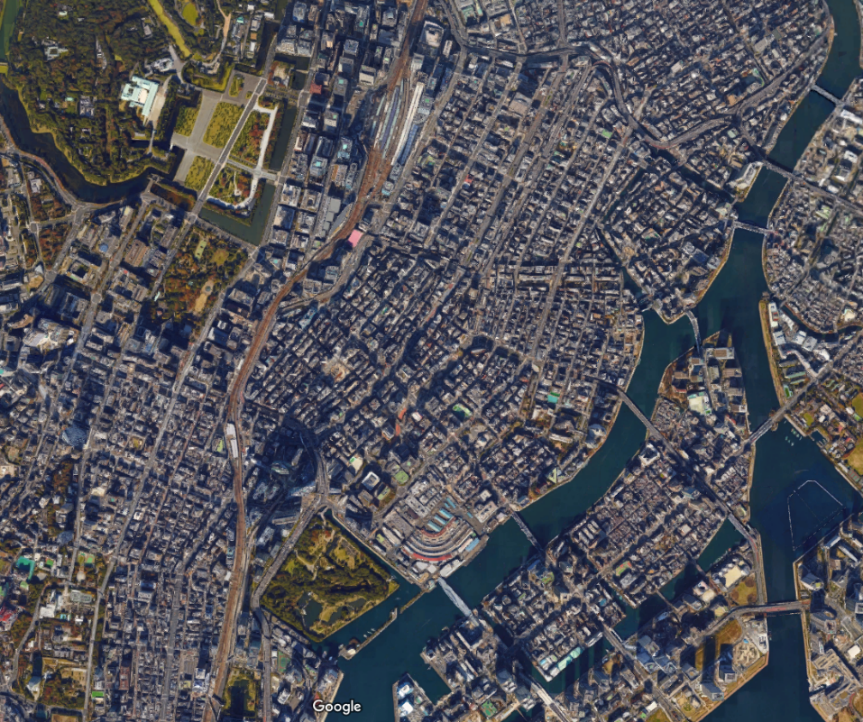


(Google; Imagery CNES/Airbus, Digital Earth Technology, Maxar Technologies, Planet.com, The GeoInformation Group, Map Data).

Salt Lake City Map


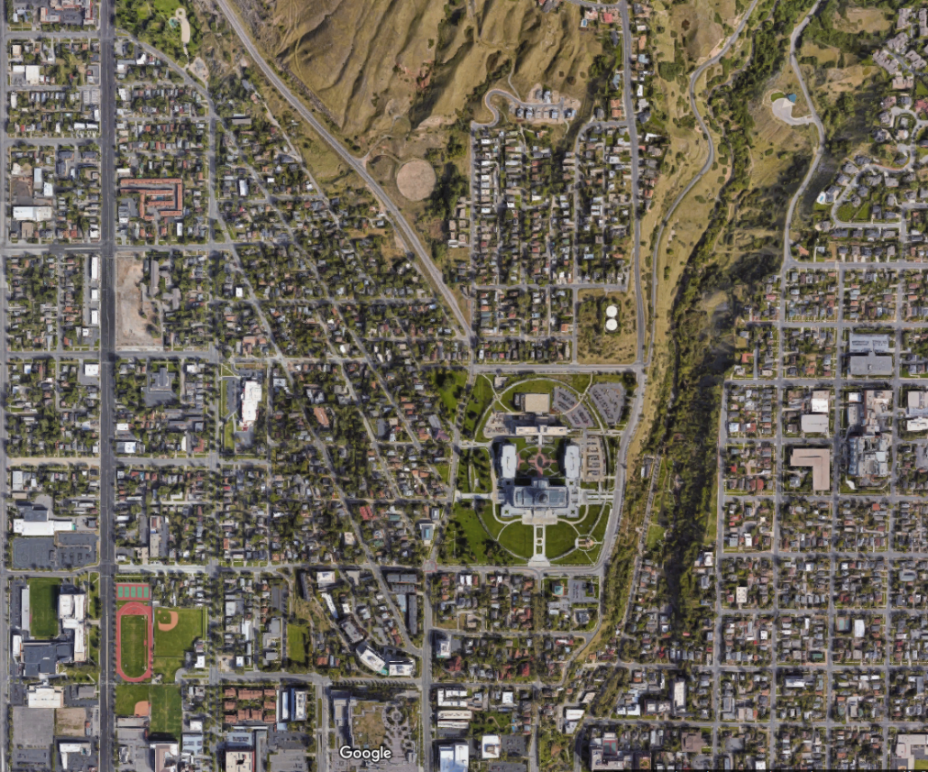


(Google; Imagery Maxar Technologies, State of Utah, USDA Farm Service Agency, Map Data).

Hamburg Map


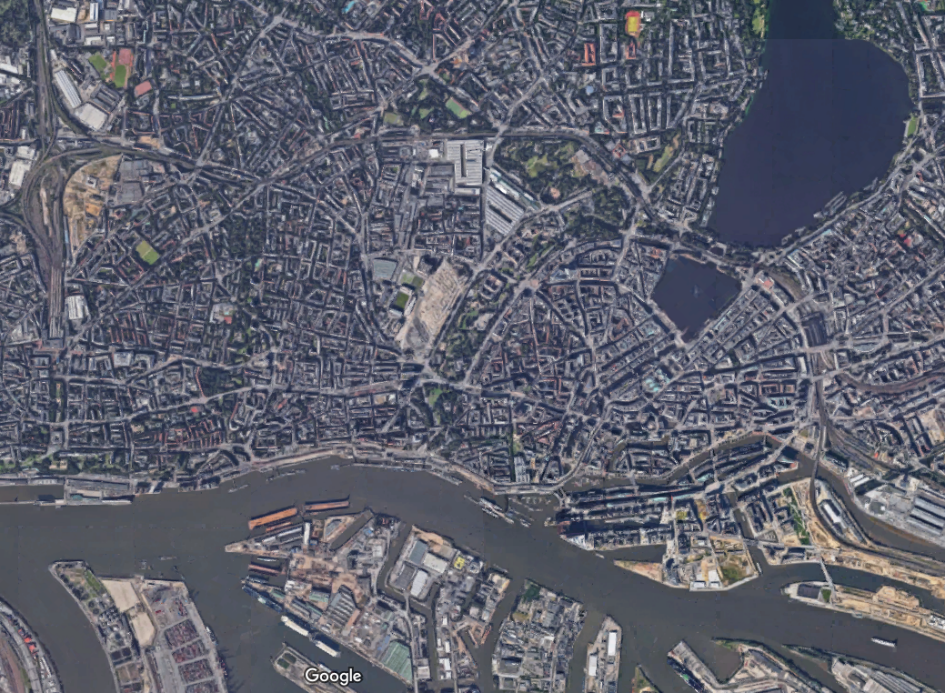


(Google; Imagery GeoBasis-DE/BKG, GeoContent, Landsat/Copernicus, Maxar Technologies Map Data).
