# Supplementary material for: Familiarity with teammate’s attitudes improves team performance in virtual reality
Source: PLoS One. 2020 Oct 26;15(10):e0241011. doi: 10.1371/journal.pone.0241011 (PMC7588115; doi:10.1371/journal.pone.0241011)
Supplement: S3 Appendix — (DOCX) [file pone.0241011.s003.docx]

S3 Appendix

**Full List of Maps and Questions**

Cape Town Map: Questions and Answers

1. How many low-income neighborhoods are on the map?
   1. 5
2. How many grocery stores are on the map?
   1. 7
3. Recently, a series of robberies have occurred in this city. Please determine the pattern to these recent robberies.
   1. Robberies are at trains stops in richer areas.
4. This city is experiencing a rise in insurgent attacks. Please determine the pattern to these recent attacks.
   1. Insurgent attacks happening at traffic circles in highly populated areas.
5. Several bombings have occurred in this city recently. Please determine the pattern of the recent bombings. Based on that pattern, where will the next one be?
   1. Bombings occurring in Gang B’s territory at Religion B churches
6. Local gangs have been establishing headquarters in this city. Please determine the current pattern of these gang HQ’s. Based on that pattern, where might another one be located?
   1. Headquarters are at School A locations that are by docks.

Tokyo Map: Questions and Answers

1. How many Bridges are on the map?
   1. 14
2. What percentage of the map is controlled by Gang A?
   1. 32.2%
3. This city has suffered from several bombings recently. Using the information on your maps, please determine the pattern of these recent bombings.
   1. Bombings occurring where there are Trash Strikes in in Gang B areas.
4. Several insurgent attacks have occurred in this area recently. Please determine the pattern of recent insurgent attacks.
   1. Attacks are near British diplomats in highly populated areas.
5. This area has recently experienced a string of robberies. Please determine the pattern of the recent robberies. Based on that pattern, where will the next one occur?
   1. Robberies occur on infrequently used roads near green space.
6. This city is having trouble with a gang who has established headquarters in the city. Please determine the pattern of the gang’s headquarters. Where might another headquarter be located based on that pattern?
   1. Headquarters are at School A locations in poorer neighborhoods.

Salt Lake City Map: Questions and Answers

1. How many churches are located in the city?
   1. 21
2. How many high-income neighborhoods are present on the map?
   1. 3
3. This area has experienced a series of robberies. Please determine the pattern of these robberies.
   1. Robberies are at recreational areas near train stops.
4. This city has experienced several insurgent attacks recently. Using the information on your maps, please determine the pattern to these attacks.
   1. Attacks are at Church A locations in richer neighborhoods.
5. This city has suffered from several bombings recently. Using the information on your maps, please determine the pattern of the recent bombings. Where might the next one occur?
   1. Bombings are at government buildings in Party C areas.
6. This area is being occupied by a certain gang. Please determine the pattern of the gang’s headquarter locations. Based on that pattern, where would another headquarter be located?
   1. Headquarters are at Church B locations in poorer areas.

Hamburg Map: Questions and Answers

1. How many Research Institutes are marked on the map?
   1. 8
2. What percentage of the map is covered by Bodies of Water?
   1. 19.3%
3. Several bombings have occurred in this city recently. Please determine the pattern of the recent bombings.
   1. They are at bus stops near bodies of water.
4. Local gangs have been establishing headquarters in this city. Please determine the current pattern of these gang headquarters.
   1. Headquarters are at recreational areas in highly populated locations.
5. This city is experiencing a rise in insurgent attacks. Please determine the pattern to these recent attacks. Based on that pattern, where will the next attack be?
   1. Attacks are at research Institutes in Party A territories.
6. Recently, a series of robberies have occurred in this city. Please determine the pattern to these recent robberies. Based on this pattern, where will the next one occur?
   1. Robberies are occurring at bus stops in Party C territories.
