# Supplementary material for: Familiarity with teammate’s attitudes improves team performance in virtual reality
Source: PLoS One. 2020 Oct 26;15(10):e0241011. doi: 10.1371/journal.pone.0241011 (PMC7588115; doi:10.1371/journal.pone.0241011)
Supplement: S4 Appendix — (DOCX) [file pone.0241011.s004.docx]

S4 Appendix

**Experience Questionnaire**

* **Video Game**

Do you or have you ever played video/computer/mobile games? (YES / NO)

a) If yes, please list the past or current games you have played most frequently:

1)______________

2) ______________

3) ______________

4) ______________

5) ______________

b) If you *currently* play games, how much time do you spend on average?

______ hours per week

c) If yes, do you play first-person shooter (FPS) games, such as Destiny or Call of Duty? (YES / NO)

If yes, how often do you play FPS games? _______ hours per week

d) If yes, do you play real-time strategy (RTS) games, such as League of Legends or Starcraft? (YES / NO)

If yes, how often do you play RTS games? _______ hours per week

***Virtual Reality**

Do you have previous experience using head-mounted virtual reality? (YES / NO)

1. If yes, how many times have you been in head-mounted virtual reality? _____ times
2. If yes, please describe how many times you have been in a head-mounted virtual reality for:
3. Research Purposes: _____
4. Gaming: ______
5. Training: ______
6. Development: _______
7. Other ____ Please Specify: _________________________
